# Pantoea agglomerans Bacteremia: A Rare Case of Bacteremia in an Immunocompetent Four-Year-Old Child

**DOI:** 10.7759/cureus.26080

**Published:** 2022-06-19

**Authors:** Hionia Haralampidou, Fani Ladomenou, Theodora Gkountoula, Panagiotis Mertzidis, Elisavet Giannousi

**Affiliations:** 1 Pediatrics, Rethymnon General Hospital, Rethymnon Crete, GRC; 2 Pediatrics, Venizeleion General Hospital, Heraklion Crete, GRC

**Keywords:** pediatrics, primary blood stream infection, immunocompetent children, bacteremia, pantoea agglomerans

## Abstract

*Pantoea agglomerans* is primarily an environmental and agricultural organism that rarely causes disease in healthy individuals. We present a case of *P. agglomerans* bacteremia in an immunocompetent four-year old boy without comorbidities who presented with fever and increased inflammatory markers. As the exact source of bacteremia could not be established, our case was considered to be an event of primary blood stream infection.

## Introduction

*Pantoea agglomerans *is a Gram negative aerobic bacillus that belongs to the *Enterobacteriaceae* family, and has previously been known as *Enterobacter agglomerans* or *Erwinia herbicol* [1-2]. It is a non-spore forming rod found in the environment, normally isolated from plant and fecal matter [1]. Although it is mainly a plant pathogen, it can be an unusual cause of human disease typically associated with thorn prick injuries or contaminated parenteral fluids. It can cause localized infections in healthy people with normal immune systems, while systemic infections are usually reported in newborns and immunocompromised hosts or patients with comorbidities [3-4]. There are also reports of secondary bacteremia or nosocomial infections that are related to medical equipment such as IV catheters or contaminated IV fluids [4]. Herein, we present a case of *P. agglomerans* bacteremia in an immunocompetent child.

## Case presentation

A previous healthy four-year-old boy presented to the emergency department (ED) with a 24-hour history of high fever associated with a sore throat, abdominal pain, and one episode of vomiting. His past medical history was unremarkable as was his family history.

On presentation to ED, the patient was febrile (T=39.1°C), with a heart rate of 150 beats per minute, a blood pressure of 117/67 mmHg, and an oxygen saturation of 98% on room air. The boy appeared unwell and tired but his physical examination was otherwise unremarkable. Laboratory investigation revealed elevated inflammatory markers with a white blood cell count of 33.8 × 103 per µL (5.0 × 103-15.5 × 103 white blood cells per µL), with a neutrophilic predominance (81.5% neutrophils, 4.9% lymphocytes) and a C-reactive protein concentration of 19.8 mg/dL (0-1 mg/dL) (Table 1).

**Table 1:** Laboratory values. WBC, white blood cell count; NEU, neutrophils; LYM, lymphocytes; MONO, monocytes; HCT, hematocrit; HGB, hemoglobin; RBC, red blood cell count; MCV, mean corpuscular volume; MCH, mean corpuscular hemoglobin; MCHC, mean corpuscular hemoglobin concentration; PLT, platelets count; CRP, C-reactive protein; ESR, erythrocyte sedimentation rate; GLU, glucose; Cr, creatinine; Na, sodium; K, potassium; SGOT, serum glutamic-oxaloacetic transaminase; SGPT, serum glutamic pyruvic transaminase

Laboratory test	Result	Reference range
WBCs (10^3^/μL)	33.8	5-15.5
NEU (%)	81.5	54-62
LYM (%)	4.9	25-33
MONO (%)	12.71	3-7
HGB (g/dL)	12.6	11.5-14.5
HCT (%)	37.9	33-43
RBC (10^6^/μL)	4.58	4-5.5
MCV (fl)	82.9	76-90
MCH (pg)	27.6	25-31
MCHC (g/dL)	33.3	32-36
PLTs (10^3^/μL)	299	150-350
ESR (mm/h)	20	0-10
CRP (mg/dl)	19.80	0-1
GLU (mg/dl)	117	60-110
Urea (mg/dl)	29	9-22.1
Cr (mg/dl)	0.36	0.20-0.43
Na (mmol/l)	134.7	135-142
K (mmol/l)	3.81	3.4-4.7
SGOT (IU/l)	25	21-44
SGPT (IU/l)	15	9-25

The urine culture was sterile and the throat swab grew only commensals. The abdominal ultrasound did not reveal any abdominal abscess or any other source of infection and the chest X-ray was negative for any infiltrates, pneumonia, or pleural effusion. With the working diagnosis of sepsis, the patient was empirically commenced on IV ceftriaxone and IV fluids. Eight hours following his admission, the blood culture grew *P. agglomerans* sensitive to ampicillin, amikacin, ceftriaxone, ciprofloxacin, cotrimoxazole, and meropenem. The identity of the organism was confirmed by mass spectrometry (Bruker MALDI Biotyper, National and Kapodistrian University of Athens). The patient responded well to treatment with ceftriaxone, which was given for 10 days in total. Repeat blood cultures were all sterile.

During the follow-up visit one month following his discharge, an immunological evaluation revealed normal immunoglobulin values, normal antibody responses, normal lymphocyte subsets and normal complement function.

## Discussion

Although the patient reported a small skin penetration by a rose thorn while playing outdoors in a rural area a week before his presentation, there was neither an identifiable source of skin infection nor any signs of soft tissue or bone infection. Thorn prick injuries are usually associated with septic arthritis and synovitis with a median time to presentation after potential injury of two weeks [5]. 

 The bacteremia in our patient could be an event of primary bloodstream infection; by definition not secondary to localized foci, as the exact source of bacteremia could not be established. However, spontaneously occurring bacteremia has been rarely reported in children, especially in those without comorbidities. There is only one report of spontaneous *P. agglomerans* bacteremia in a child with sepsis after rotavirus gastroenteritis [6]. In that instance, it was postulated that the preceding gastrointestinal insult facilitated bacterial translocation across the gut mucosa. There is also one report of *P. agglomerans* bacteremia secondary to a urinary tract infection episode in a child with posterior urethral valves [7]. It is noteworthy that these are the only two reports of *P. agglomerans* bacteremia in immunocompetent children. However, our patient differs from the previous cases as he had no preceding infection.

In concordance with previous literature findings, bacteremia appeared to be transient and did not recur during therapy, and an antibiotic course of 10 days seemed to be curative. Regarding the effective antibacterial treatment, antimicrobial susceptibility has been tested in 53 pediatric cases with *P. agglomerans* infections [8]. Similar to our patient, all isolates were uniformly susceptible to amikacin, gentamicin, meropenem, and trimethoprim-sulfamethoxazole. In addition, 92.5% of the isolates were susceptible to broad-spectrum cephalosporins and semisynthetic penicillins, 62.3% to extended-spectrum cephalosporins, and 47.2% to ampicillin [8]. 

*Pantoea agglomerans* may cause serious morbidity and mortality, especially in young patients with underlying comorbidities. The outcome in our case was benign probably because of the early diagnosis, appropriate treatment, and absence of comorbidities and immunodeficiency. This large spectrum of clinical manifestations may result from the different characteristics between community and hospital-acquired cases of *P. agglomerans* infections [7].

## Conclusions

*Pantoea agglomerans *is an uncommon cause of infection in children, with a large spectrum of clinical manifestations. To the best of our knowledge, this is the first case of spontaneous* P. agglomerans *bacteremia in an immunocompetent child without comorbidities and preceding infections.
